# High prevalence of SARS-CoV-2 infection among symptomatic healthcare workers in a large university tertiary hospital in São Paulo, Brazil

**DOI:** 10.1186/s12879-020-05662-8

**Published:** 2020-12-02

**Authors:** Carolina Palamin Buonafine, Beatriz Nobre Monteiro Paiatto, Fabyano Bruno Leal, Samantha Faria de Matos, Camila Ohomoto de Morais, Giovanna Guazzelli Guerra, Marcus Vinicius Vidal Martuchelli, Danielle Bruna Leal Oliveira, Edison Luiz Durigon, Camila Pereira Soares, Erika Donizette Candido, Bruna Larotonda Telezynski, Marco Aurélio Palazzi Sáfadi, Flávia Jacqueline Almeida

**Affiliations:** 1 Department of Pediatrics, Santa Casa de São Paulo School of Medical Sciences, Rua Dr. Cesário Motta Jr., 61, São Paulo, SP 01221-020 Brazil; 2 Department of Microbiology, Laboratory of Molecular and Clinical Virology, Institute of Biomedical Sciences - University of São Paulo, São Paulo, SP Brazil; 3 Department of Pediatrics, Division of Infectious Diseases, Santa Casa de São Paulo Hospital, São Paulo, SP Brazil

**Keywords:** SARS-CoV-2, COVID-19, Health care workers, Epidemiology, Risk factors

## Abstract

**Background:**

Brazil became the epicenter of the COVID-19 pandemic in Latin America since May 2020, reporting the highest number of cases and deaths in the region. Healthcare workers (HCWs) are at increased risk of SARS-CoV-2 infection, experiencing a significant burden from COVID-19. Identifying and understanding the clinical characteristics and risk factors associated with infection are of paramount importance to inform screening strategies and infection control practices in this scenario. The aims of this study were to investigate the prevalence and clinical characteristics of HCWs with COVID-19 symptoms.

**Methods:**

Between March 21st and May 22nd, 2020 a cross-sectional study was performed in a tertiary university hospital in São Paulo. Prevalence of SARS-CoV-2 infection among HCWs with COVID-19 symptoms was determined by RT-PCR testing on nasopharyngeal and oropharyngeal samples. Participants were asked to complete an electronic structured questionnaire including clinical and demographic data.

**Results:**

Overall, 125 (42.37%) of 295 symptomatic HCWs tested positive for SARS-CoV-2. Over the 10-week study period, positivity rates varied from 22.2% (95% CI 15.9–60.3%) in the second week to 55.9% (95% CI 43.2–68.6%) in the sixth week, reaching a plateau (38–46%) thereafter. Median (SD) age was 34.2 (9.9) years and 205 (69.5%) were female. We did not find significant differences in the prevalence of the most commonly reported underlying medical condition among healthcare workers that tested positive or negative for SARS-CoV-2 infection. After multivariable analysis, using logistic regression, anosmia (adjusted OR 4.4 95% CI 2.21–8.74) and ocular pain (adjusted OR 1.95 95% CI 1.14–3.33) were the only symptoms independently associated with positivity for SARS-CoV-2 infection. Follow-up information on clinical outcomes showed that 9 (7.2%) HCWs were hospitalized (seven were male) and 2 (1.6%) died.

**Conclusions:**

The findings of this study confirmed the high burden of SARS-CoV-2 infection among healthcare workers in the hardest hit city by the pandemic in Latin America. Anosmia and ocular pain were symptoms independently associated with COVID-19 diagnosis. In low and middle-income countries, where limited availability of tests is frequent, these findings may contribute to optimize a targeted symptom-oriented screening strategy.

## Background

Since the emergence of the Severe Acute Respiratory Syndrome Coronavirus 2 (SARS-CoV-2) in China, in December 2019, the coronavirus disease 2019 (COVID-19) has affected almost 38 million people from 214 countries and territories around the world [1]. In Brazil, the hardest hit country in Latin America, since February 25, 2020, approximately 5 million cases and 150,000 SARS-CoV-2-associated deaths were confirmed by October [2].

Healthcare workers (HCWs) are at increased risk of healthcare-associated infections, due to the frontline nature of their work. According to WHO, during the SARS epidemic, in 2002–2003, rates as high as 20% of all persons affected were HCWs [3].

Although several studies already investigated the epidemiology of and risk factors for SARS-CoV-2 infection among HCWs in high-income countries, there is a lack of data from low and middle-income countries, where shortage of personal protective equipment (PPE), diagnostic tests and other vital supplies represent one of the most urgent challenges faced by public health systems [4–13].

Identifying and understanding the clinical characteristics, outcomes and risk factors associated with SARS-CoV-2 infection among HCWs are of paramount importance to mitigate the spread of the virus in the hospital setting, for high risk patients and other HCWs. This information will be critical to inform screening strategies and infection control practices, particularly in places experiencing challenging scenarios, with high burden of disease and limited resources and protective supplies.

In this context, we aimed to estimate the prevalence and the clinical presentations of SARS-CoV-2 infection among symptomatic HCWs from a tertiary university hospital during the pandemic in São Paulo, Brazil, the current epicenter of the COVID-19 pandemic in Latin America.

## Methods

Between March 21st and May 22nd, 2020, HCWs from Santa Casa de São Paulo Hospital were defined as symptomatic and invited to participate in the study if presented with self-reported fever or any of the following: acute respiratory symptoms (cough, nasal congestion, sore throat, shortness of breath), loss or changed sense of smell or taste, ocular symptoms, headache, arthralgia, myalgia, fatigue, diarrhea, nausea, and vomiting. HCWs were recruited as part of a research project with advertising warnings in diverse areas of the hospital.

Santa Casa de São Paulo Hospital (SCSP) is a 600-bed university hospital, with primary, secondary, and tertiary care facilities and 4597 HCWs, of which 1902 (41.3%) are nurses, 1298 (28.3%) physicians (including the residents) and 1397 (30.4%) administrative staff.

An electronic questionnaire (supplementary file 1), developed for this study, was performed, including information on clinical and demographic data, with a 15-day follow-up after onset of symptoms (supplementary file 2).

Nasopharyngeal and oropharyngeal swabs in 3 ml saline 0,9% were collected [2] and sent daily to the Laboratory of Clinical and Molecular Virology (LVCM) of the University of Sao Paulo-Brazil for molecular testing. The real time reverse transcriptase–polymerase chain reaction (RT-PCR) tests for SARS-CoV-2 were carried out using assays developed at the Charité (Institute of Virology, University of Berlin, Germany) and modified by LVCM [8].

Samples were collected between the second and the seventh day after the symptom’s onset and 50 μl viral nucleic acid was obtained from 400 μl of swabs out on the NucliSens easyMag® platform fully automated (BioMerieux, Lyon, France).

Study approval was given by the Ethics Committee of SCSP and written informed consent was obtained from all participants.

### Statistical analysis

Characteristics of the study population were presented in counts and percentages for qualitative variables and with means ± standard deviations (SD) for quantitative variables.

The Chi-square test and the contingency table were used to verify the possible association with the qualitative variables. A significance level of 5% was established (*p* < 0.05). To assess the distribution of the quantitative variables, we used the Shapiro-Wilk test. For the quantitative variables that were not normally distributed, the non-parametric Mann-Whitney U test was used. We used Stata Statistics Software version 13.1 for this analysis.

Multivariable analysis using logistic regression compared qualitative variables (variables with *P* < 0.20 in the univariable analysis were considered in the pre-selection). We used a Stepwise Forward method. For this analysis, we used the SPSS program version 13.0.

## Results

In the period of the study, between March 21st and May 22nd, 2020, a total of 295 symptomatic HCWs were tested for SARS-CoV-2, of whom 125 (42.37%) were found positive.

**Fig. 1:**
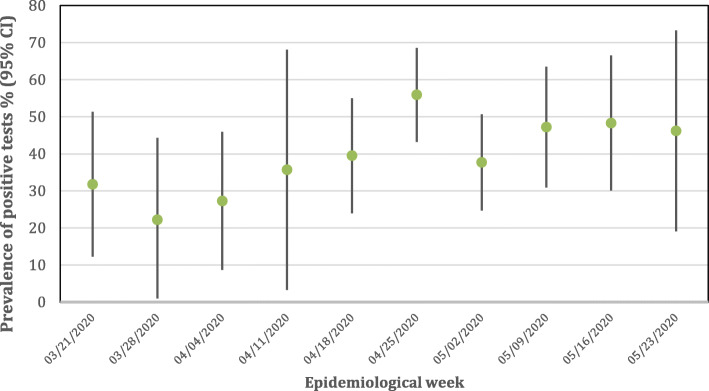
Weekly positivity rate (95% CI) of SARS-CoV-2 test results among symptomatic health care workers

Over the 10-week study period, positivity rates varied from 22.2% (CI 95 15.9–60.3%) in the second week to rates as high as 55.9% (CI 95 43.2–68.6%) in the sixth week, reaching a plateau (38–46%) in the following weeks (Fig. 1).

Among the 295 HCWs included, 163 (55.3%) were physicians (residents, fellows, and assistants), 105 (35.6%) from the nursing staff, and the remaining 27 (6.4%) were physiotherapists, radiology technicians and others.

The median age was 34.2 years (SD 9.9); 130 (44%) were younger than 30 years of age, and 205 (69.5%) were female. The average time between the onset of the symptoms and the RT-PCR testing was 6.3 days (SD 4.1). Presence of known underlying medical conditions was identified in 57 (19.3%) HCWs, of which 23 (7.8%) were persons with chronic lung disease or asthma, 12 (4%) metabolic disorders including diabetes mellitus, 9 (3%) with cardiovascular diseases and 15 (5%) obesity with BMI ≥ 40. Only 7 (2.3%) were individuals over 60 years old (Table 1).

**Table 1:** Demographic Characteristics of symptomatic HCWs tested for SARS-CoV-2 in Sao Paulo (*N* = 295)

Variable	All HCWs ***N*** = 295 (100%)	SARS-CoV-2 Positive ***N*** = 125 (42.4%)	SARS-CoV-2 Negative ***N*** = 170 (57.6%)	***P*** value
**Sex**				0.002
Male, n (%)	90 (30.5%)	50 (40%)	40 (23.6%)	
Female, n (%)	205 (69.5%)	75 (60%)	130 (76.4%)	
**Age, mean (SD) years**	34.2 (9.9)	34.5 (9.9)	34 (9.9)	0.81
**Days of symptoms mean (SD)**	6.3 (4.1)	6 (3.8)	6.5 (4.2)	0.56
**Underlying medical Conditions, n (%)**	57 (19.3%)	21 (16.8%)	36 (15.1%)	0.34
Chronic lung disease or asthma	23 (7.8%)	9 (7.2%)	14 (8.2%)	0.91
Diabetes mellitus	12 (4%)	4 (3.2%)	8 (4.7%)	0.56
Cardiovascular disease	9 (3%)	4 (3.2%)	5 (2.9%)	0.98
Obesity (BMI ≥ 40)	14 (4.7%)	6 (4.8%)	8 (4.7%)	0.73
**Age > 60 years, n (%)**	7 (2.3%)	1 (0.8%)	6 (3.5%)	0.24
**Professional category, n (%)**				0.32
Medical team	163 (55.3%)	69 (55.2%)	94 (55.2%)	
Nursing team	105 (35.6%)	48 (38.4%)	57 (33.5%)	
Other	27 (9.2%)	8 (6.4)	19 (10.7%)	
**Medical Specialists, n (%)**				
General Practice	56 (34.3%)	25 (36.2%)	31 (32.9%)	0.7
Pediatrics	38 (23.3%)	15 (21.7%)	23 (24.5%)	0.69
Surgery	23 (14.1%)	10 (14.4%)	13 (13.8%)	0.91
Otorhinolaryngology	13 (7.9%)	7 (10.1%)	6 (6.4%)	0.39
Gynecology and Obstetrics	9 (5.5%)	4 (5.7%)	5 (5.3%)	> 0.99
**Close contact with confirmed COVID-19, n (%)**	218 (73.9%)	91 (72.8%)	127 (74%)	0.79
**Total, n**	**295**	**125**	**170**	

*BMI* Body Mass Index, *COVID-19* Coronavirus Disease, *HCW* Health Care Worker, *SARS-CoV-2* Severe Acute Respiratory Syndrome Coronavirus 2

The median age of the HCWs that tested positive for SARS-CoV-2 was similar to the group that tested negative, (34.5 and 34 years, respectively). A higher proportion of male HCWs was found among HCWs who tested positive for SARS-CoV-2 (40% vs 23.6%. *p* = 0.002). We did not find significant differences in the prevalence of the most commonly reported underlying medical condition among HCWs that tested positive or negative for SARS-CoV-2 infection: chronic lung disease or asthma (7.2% vs 8.2%. *p* = 0.91), metabolic disorders including diabetes mellitus (3.2% vs 4.7%. *p* = 0.56), cardiovascular diseases (3.2% vs 2.9%. *P* = 0.98), obesity with BMI ≥ 40 (4.8% vs 4.7%. *p* = 0.73). The proportion of individuals over 60 years was also similar in both groups (0.8% vs 3.5%. *p* = 0.24). There was no association for testing positive with profession, medical specialty or reporting contact with a confirmed case of COVID-19.

The most frequent symptoms reported among HCWs who tested positive and negative for SARS-CoV-2, respectively, were headache (92.8 and 90.5%. *p* = 0.5), nasal congestion (88.8 and 87%. *P* = 0.76), cough (85,6 and 85%. *P* = 0.94), fatigue (89.6 and 81.7%. *p* = 0.06) and myalgia (84 and 78.2%. *p* = 0.21).

Among the symptoms reported by the HCWs, fever and anosmia were statistically associated with positivity for SARS-CoV-2 infection (Table 2).

**Table 2:** Association of Symptoms with SARS-CoV-2 positivity among symptomatic HCWs in Sao Paulo. (*N* = 295)

Variable	All HCWs ***N*** = 295 (100%)	SARS-COV-2 Positive ***N*** = 125 (42.4%)	SARS-COV-2 Negative ***N*** = 170 (57.6%)	***P*** value
**Symptoms, n (%)**				
Headache	270 (91.5%)	116 (92.8%)	154 (90.5%)	0.5
Nasal congestion	260 (88.1%)	111 (88.8%)	149 (87%)	0.76
Cough	252 (85.4%)	107 (85.6%)	145 (85%)	0.94
Fatigue	251 (85%)	112 (89.6%)	139 (81.7%)	0.06
Myalgia	238 (80.6%)	105 (84%)	133 (78.2%)	0.21
Sore throat	230 (77.9%)	96 (76.8%)	134 (78.8%)	0.67
Chills	224 (75.9%)	101 (80.8%)	123 (72.3%)	0.09
Ocular pain	194 (65.7%)	90 (72%)	104 (61.1%)	0.05
Fever	208 (70.5%)	96 (76.8%)	112 (65.8%)	0.04
Arthralgia	173 (58.6%)	81 (64.8%)	92 (54.1%)	0.06
Diarrhea	165 (55.9%)	69 (55.2%)	96 (56.4%)	0.82
Abdominal pain	151 (51.1%)	68 (54.4%)	83 (48.8%)	0.3
Shortness of breath	143 (48.4%)	69 (53.6%)	74 (44.1%)	0.17
Cutaneous rash	126 (42%)	58 (46.4%)	68 (40%)	0.27
Anosmia	50 (16.9%)	35 (28%)	15 (8.8%)	0.001
**Total, n**	**295**	**125**	**170**	

*HCW* Health Care Worker, *SARS-CoV-2* Severe Acute Respiratory Syndrome Coronavirus 2

In the univariable analysis for risk factors associated with SARS-CoV-2 positivity, male sex, anosmia, and fever were statistically significant (Table 3). After multivariable logistic regression analysis, male sex (adjusted OR 2.13, 95% CI 1.26–3.61), anosmia (adjusted OR 4.40, 95% CI 2.21–8.74), and ocular pain (adjusted OR 1.95 95% CI 1.14–3.33) were associated with positivity for SARS-CoV-2 infection.

**Table 3:** Univariable and multivariable analysis regarding sex, age and symptoms associated with SARS-CoV-2 positivity

Variable	Univariable analysis OR (95% CI)	***P*** value	Multivariable analysis Adjusted OR (95% CI)	***P*** value
Age > 60 years	0.22 (0.02–1.85)	0.24		
Sex - Male	2.17 (1.31–3.59)	0.002	2.13 (1.26–3.61)	0.005
Anosmia	4.02 (2.08–7.76)	0.001	4.40 (2.21–8.74)	< 0.001
Ocular pain	1.63 (0.99–2.68)	0.05	1.95 (1.14–3.33)	0.01
Fever	1.71 (1.02–2.89)	0.04		0.24
Shortness of breath	1.40 (0.86–2.27)	0.17		0.66
Fatigue	1.92 (0.96–3.85)	0.06		0.14
Arthralgia	1.56 (0.97–2.51)	0.06		0.57
Chills	1.61 (0.92–2.81)	0.09		0.30
Myalgia	1.46 (0.80–2.66)	0.21		
Cutaneous rash	1.30 (0.81–2.07)	0.27		
Abdominal pain	1.27 (0.80–2.02)	0.30		
Headache	1.34 (0.57–3.14)	0.50		
Sore throat	0.89 (0.51–1.55)	0.67		
Nasal Congestion	1.12 (0.54–2.29)	0.76		
Diarrhea	0.95 (0.60–1.51)	0.82		
Cough	1.02 (0.53–1.97)	0.94		

*OR* Odds ratio, *CI* Confidence interval

Among the 125 HCWs that tested positive for SARS-CoV-2 infection, follow-up information on clinical outcomes showed that 9 (7.2%) were hospitalized (seven were male) and 2 (1.6%) died. Although none of the 9 hospitalized COVID-19 HCWs were aged > 60 years, their median age (41.8 y) was higher than the median age of the COVID-19 HCWs that were not hospitalized (34.2 y). The first death was a 37-year old male, with a BMI ≥ 40. The second death was a 38-year old male with BMI > 40, hypertension and severe asthma (Table 4).

**Table 4:** Hospitalizations and deaths, by age group among health care workers with COVID-19

Age group (number of cases)	Hospitalization Number (%)	Death Number (%)
< 40 y (81)	3 (3.7)	2 (2.4)
40–60 y (37)	6 (16.2)	0
> 60 y (7)	0	0
Total (125)	9 (7.2)	2 (1.6)

## Discussion

Our results, showing a high prevalence of SARS-CoV-2 infection among HCWs, are in line with previous data that demonstrated that HCWs have higher susceptibility to respiratory infections. These findings were also observed in other zoonotic coronavirus outbreaks (SARS and MERS), when a substantial proportion of the infected population were HCWs [3, 9]. They are repeatedly exposed to COVID-19 patients, particularly those working on frontline, where certain procedures (intubation, contact with secretions, aerosol-generating procedures) increase the risk of infection, highlighting the importance of using the recommended personal protective equipment (PPE) measures [14].

In our hospital, universal use of masks by all HCWs was implemented as a standard practice in the hospital only in the first week of May. According to the standard infection prevention protocol in place at that time, PPE was recommended only when caring for suspected or confirmed COVID-19 patients. It is important to emphasize that during the whole study period, availability and access to PPE, as well as training and supervision from the infection prevention and control committee members, were fully provided to HCWs, with adequate adherence to the protocols. Hospitalized patients were in transmission-based isolation precautions only when presenting symptoms compatible with COVID-19 or with history of known exposure to a COVID-19 patient in the previous 14 days. Although HCWs are at higher risk of SARS-CoV-2 infection at work, while caring for suspected or confirmed COVID-19 patients, household and community transmission are also relevant, particularly during the period of the study, when an intense activity of community transmission was occurring in São Paulo (In May more than 200 new COVID-19 cases per 100,000 persons within 14 days was reported) [15].

COVID-19 has a wide spectrum of clinical manifestations ranging from asymptomatic illness to severe cases with multi-organ failure and death [10]. The rates of hospitalization among HCWs with COVID-19 (7.2%), as well as case fatality rates (1.6%) found in our study are similar to those reported in US among HCWs patients with data available on age and health outcomes (respectively 8 and 0.6%) [16]. Interestingly, these rates are lower than those found in non-HCWs population with COVID-19 in Brazil [17], probably reflecting the younger median age of the HCWs of our hospital. Furthermore, it is likely that among HCWs the level of suspicion to the disease symptoms is higher, enabling them to an earlier diagnosis and treatment, which may improve COVID-19 outcomes, and identifying a higher proportion of mild cases. Similar to earlier findings [18], our data also showed that male sex was associated with a higher risk of severe outcomes (78% of the HCWs that were hospitalized and both that died were men).

There are conflicting results in the literature to identify the job category with the highest risk of COVID-19 among HCWs [6, 7, 11]. In our study, physicians represented the majority of the cases, even though nursing staff form the largest subset of employees, representing 41.3% of the HCWs in the hospital. Compared to other HCWs, physicians seem to have greater awareness of disease symptoms, facilitating their access to testing and medical care.

In the city of São Paulo, the epicenter of the pandemic in Latin America, on June 14th, during the study period, there were 98,000 confirmed cases of COVID-19, of which, 1902 (1.94%) were HCWs, with 26 confirmed deaths (CFR of 1.3%), similar to the CFR found in our study [15]. Data from China showed that a total of 3387 of 77,262 patients with COVID-19 (4.4%) were HCWs, with 23 deaths (CFR of 0.67%) [19].

The sustained high weekly prevalence rates of SARS-CoV-2 infection among symptomatic HCWs observed in our study (from 22.3 to 55.9%), when compared to similar studies from Asia, Europe and US [11–14, 20–22] is concerning (Fig. 1). The majority of our cohort had a mild illness, which could potentially represent a risk of continued routine of working throughout the illness, facilitating the transmission of the virus into the hospital to patients and other HCWs. It is also concerning the long median time between symptom onset and RT-PCR testing among HCWs found in our study (6.3 days), despite the presence of recommendations to self-isolation when symptomatic. The working overload and the limited number of HCWs during the peak of the pandemic, together with a lack of access to immediate testing outside the protocol during the study period in our hospital, are potential issues contributing for the long median time after symptom onset reported by HCWs when they were tested. This finding may represent a significant risk of increasing transmission. Similar studies in Europe demonstrated that a high proportion of HCWs maintained their work routine in the hospital even presenting mild symptoms [11–13]. At the time the study was conducted, the official policy in the hospital recommended that symptomatic HCWs should be immediately excluded from the workplace for a minimum of 14 days. Although HCWs in the hospital were instructed and trained, the results of our study highlight the importance of having not only well-stablished guidance on the use of PPE as well as clear recommendations on sick leave policies for all HCWs with suspected COVID-19, but also strong supervision for compliance.

Limitations of this study include the single-hospital design and testing only symptomatic HCWs. The increased awareness of COVID-19 symptoms among physicians, facilitating their access to testing and medical care, comparing to other HCWs, contributed to a reporting bias, leading to an overrepresentation of this category in our study population. Furthermore, the study was not designed to identify the source of infection among symptomatic HCWs. This approach limited the possibility of a better understanding on the transmission dynamics as well as the true prevalence of SARS-Co-V-2 infection among HCWs. However, to our knowledge this is the first report describing prevalence, clinical characteristics, and outcomes of SARS-CoV-2 infection among HCWs in Latin America.

One of the great challenges we faced in Brazil, and probably one of the reasons that contributed to the high burden of COVID-19 in the country, was the limited availability of virologic testing. Only suspected cases that were hospitalized could be tested for SARS-CoV-2 in the public health system. During the initial phase of the pandemic, even symptomatic HCWs were not able to be tested for the virus in our hospital as well as several others in the country. These limited testing clearly compromises the strategies to contain nosocomial transmission of the virus to inpatients and to other HCWs [23]. Expanding capacity of testing among HCWs, including not only symptomatic, but also asymptomatic (facilitating detection of those that are in the presymptomatic phase, when transmission is already occurring) is the logical strategy in places where budget-resource constraints are not present, particularly among groups like HCWs, susceptible to high exposure to infected patients. Recent data from a large UK teaching hospital demonstrated the value of a comprehensive screening, including asymptomatic and oligosymptomatic HCWs, emphasizing the importance of this expanded strategy for protecting patients and hospital staff [24].

## Conclusions

The findings of this study confirmed the high burden of SARS-CoV-2 infection among healthcare workers in the hardest hit city by the pandemic in Latin America and provides valuable information on symptoms in the early phase of COVID-19. Anosmia and ocular pain were symptoms independently associated with COVID-19 diagnosis. In low and middle-income countries, where limited availability of tests is frequent, these findings may contribute to optimize a targeted symptom-oriented screening strategy.

## Supplementary Information

**Additional file 1: Supplementary file 1**. Questionnaire. Questionnaire with data on demographical, clinical, and epidemiological information.

**Additional file 2: Supplementary file 2**. Follow-Up Questionnaire. Questionnaire completed after 15 days since the onset of symptoms, with information on symptoms and duration, including interventions, for HCWs who tested Positive for SARS-CoV-2.

## Data Availability

The datasets used and/or analyzed during the current study are available from the corresponding author on reasonable request.

## Abbreviations

BMI: Body Mass Index
COVID-19: Coronavirus disease 2019
HCWs: Healthcare workers
LVCM: Laboratory of Clinical and Molecular Virology
MERS: Middle East Respiratory Syndrome
RT-PCR: Real time reverse transcriptase–polymerase chain reaction
PPE: Personal protective equipment
SARS: Severe Acute Respiratory Syndrome
SARS-CoV-2: Severe acute respiratory syndrome coronavirus 2
SCSP: Santa Casa de São Paulo Hospital
SD: Standard Deviation
