# Supplementary material for: High prevalence of SARS-CoV-2 infection among symptomatic healthcare workers in a large university tertiary hospital in São Paulo, Brazil
Source: BMC Infect Dis. 2020 Dec 2;20:917. doi: 10.1186/s12879-020-05662-8 (PMC7709093; doi:10.1186/s12879-020-05662-8)
Supplement: Supplementary file 1 — Additional file 1: Supplementary file 1. Questionnaire. Questionnaire with data on demographical, clinical, and epidemiological information. [file 12879_2020_5662_MOESM1_ESM.docx]

**Questionnaire - SARS-CoV-2 prevalence study**

Medical students, medical doctors, nurses, and other health care workers are at a disproportional high and constant risk for influenza, SARS-CoV-2, and other respiratory infections.

The aims of this study were to investigate the prevalence of SARS-CoV-2 infection and clinical characteristics of healthcare workers with ILI and COVID-19 symptoms from the Santa Casa de São Paulo Hospital. The secondary objective is to analyze the prevalence of annual immunization against influenza among HCWs.

Advisors: Dr Flavia Jacqueline Almeida and Dr. Marco Aurelio Sáfadi
Investigator: Beatriz Nobre Monteiro Paiatto and Carolina Palamin Buonafine

1. **Demographical and epidemiological information**
2. Sex

- Female
- Male

1. Age
2. Occupation/Profession
3. Do you have any of the following conditions? You can check more than one option.

- Pregnancy
- More than 60 years
- Use of aspirin
- Chronic lung disease (including asthma)
- Tuberculosis
- Cardiovascular disease (including hypertension)
- Kidney disease
- Liver disease
- Blood disorder
- Metabolic disorder (including Diabetes Mellitus)
- Immunosuppressive therapy
- Obesity with BMI ≥ 40

1. Check the year(s) that you were vaccinated for Influenza.

- 2020
- 2019
- 2018
- 2017
- 2016
- 2015

1. If you were vaccinated this year (2020) , where did you take it?

- Medical Care Service for students
- Medical Care Service for health care workers
- Health Public Center
- Private Clinic
- Other

1. Did you have contact with a confirmed case of COVID-19 in the last 14 days?

- Yes
- No

1. Did you have contact with a suspect case of COVID-19 in the last 14 days?

- Yes
- No

1. Did you travel abroad until 14 days before the onset of your symptoms?

- Yes
- No

1. How long have passed since your symptoms first appeared?

- Today
- 1 day
- 2 days
- 3 days
- 4 days
- 5 days
- 7 days
- 14 days
- More than 14 days

1. **Clinical information (Symptoms)**
2. Check the symptom(s) that you had/have.

- Cough
- Nasal congestion
- Chills
- Headache
- Myalgia
- Sore throat
- Arthralgia
- Fatigue
- Abdominal pain
- Cutaneous rash
- Diarrhea
- Ocular pain
- Anosmia
- Shortness of breath

1. Please, write below other symptoms not listed.
2. **Knowledge about disease**
3. Once aware that you are infected with influenza, would you go to work/study?

- Yes
- No

1. Once aware that you are infected with SARS-CoV-2, would you go to work/study?

- Yes
- No

1. Do you think that the causative agent of your symptoms is the influenza virus?

- Yes
- No

1. Do you think that the causative agent of your symptoms is the SARS-CoV-2 virus?

- Yes
- No

1. Did you take any medicine? Including antipyretic, anti-inflammatory and analgesic.

- Yes
- No

1. Do you intend to take any medicine if you a have a positive test for COVID-19?

- Yes
- No

1. If yes, which one?
2. If you have a positive test, do you authorize us to contact you by e-mail or Whatsapp? If yes, please write your phone number or e-mail.
3. **Test Results**
4. Influenza test

- Positive
- Negative
- Pending

1. SARS-CoV-2 test

- Positive
- Negative
- Pending
