# Supplementary material for: High prevalence of SARS-CoV-2 infection among symptomatic healthcare workers in a large university tertiary hospital in São Paulo, Brazil
Source: BMC Infect Dis. 2020 Dec 2;20:917. doi: 10.1186/s12879-020-05662-8 (PMC7709093; doi:10.1186/s12879-020-05662-8)
Supplement: Supplementary file 2 — Additional file 2: Supplementary file 2. Follow-Up Questionnaire. Questionnaire completed after 15 days since the onset of symptoms, with information on symptoms and duration, including interventions, for HCWs who tested Positive for SARS-CoV-2. [file 12879_2020_5662_MOESM2_ESM.docx]

**Follow-Up for HCWs who tested Positive for SARS-CoV-2**

This is a questionnaire to be completed after 15 days since the onset of symptoms.

1. **Symptoms and duration**
2. How long did fever last?

- 1 day
- 2 days
- 3 days
- 4 days
- 5 days
- 6 days
- 7 days
- 7 – 14 days
- More than 14 days
- Did not have this symptom

1. How long did sore throat last?

- 1 day
- 2 days
- 3 days
- 4 days
- 5 days
- 6 days
- 7 days
- 7 – 14 days
- More than 14 days
- Did not have this symptom

1. How long did nasal congestion last?

- 1 day
- 2 days
- 3 days
- 4 days
- 5 days
- 6 days
- 7 days
- 7 – 14 days
- More than 14 days
- Did not have this symptom

1. How long did cough last?

- 1 day
- 2 days
- 3 days
- 4 days
- 5 days
- 6 days
- 7 days
- 7 – 14 days
- More than 14 days
- Did not have this symptom

1. How long did headache last?

- 1 day
- 2 days
- 3 days
- 4 days
- 5 days
- 6 days
- 7 days
- 7 – 14 days
- More than 14 days
- Did not have this symptom

1. How long did myalgia last?

- 1 day
- 2 days
- 3 days
- 4 days
- 5 days
- 6 days
- 7 days
- 7 – 14 days
- More than 14 days
- Did not have this symptom

1. How long did arthralgia last?

- 1 day
- 2 days
- 3 days
- 4 days
- 5 days
- 6 days
- 7 days
- 7 – 14 days
- More than 14 days
- Did not have this symptom

1. How long did diarrhea last?

- 1 day
- 2 days
- 3 days
- 4 days
- 5 days
- 6 days
- 7 days
- 7 – 14 days
- More than 14 days
- Did not have this symptom

1. How long did ocular pain last?

- 1 day
- 2 days
- 3 days
- 4 days
- 5 days
- 6 days
- 7 days
- 7 – 14 days
- More than 14 days
- Did not have this symptom

1. How long did anosmia last?

- 1 day
- 2 days
- 3 days
- 4 days
- 5 days
- 6 days
- 7 days
- 7 – 14 days
- More than 14 days
- Did not have this symptom

1. How long did shortness of breath last?

- 1 day
- 2 days
- 3 days
- 4 days
- 5 days
- 6 days
- 7 days
- 7 – 14 days
- More than 14 days
- Did not have this symptom

1. Did new symptom(s) appear?

- Yes
- No

1. In affirmative case, please, describe which symptom(s) and the duration.
2. **Support measures**
3. Did you develop any complication?

- Yes
- No

1. In affirmative case, please, describe which complication.
2. Did you need to be hospitalized?

- Yes
- No

1. In affirmative case, how long were you hospitalized?
2. Did you take antibiotic?

- Yes
- No

1. In affirmative case, which antibiotic was taking? For how long did you take?
2. Did you take Oseltamivir?

- Yes
- No

1. Did you take any other medications (except antipyretics)?

- Yes
- No

1. In affirmative case, which medication?
